# Editorial: Epigenetic regulation of genome integrity and its implications in human diseases

**DOI:** 10.3389/fcell.2024.1535839

**Published:** 2024-12-10

**Authors:** Zhiming Li, Xiao Chen, Chun-Long Chen

**Affiliations:** ^1^ West China School of Public Health and West China Fourth Hospital, State Key Laboratory of Biotherapy, Sichuan University, Chengdu, China; ^2^ Marine College, Shandong University, Weihai, China; ^3^ Suzhou Research Institute, Shandong University, Suzhou, China; ^4^ Institut Curie, PSL Research University, CNRS UMR3244, Dynamics of Genetic Information, Sorbonne Université, Paris, France

**Keywords:** epigenetic regulation, histone modification, DNA methylation, RNA modification, genome integrity, cancer epigenetics, epigenetic inheritance

The epigenetic paradigm encompasses a vast network of factors that form the building blocks and regulatory core of the eukaryotic chromatin. Among the key determinants of epigenetic regulation, covalent chromatin modifications, including histone modifications and DNA methylation, histone variants, RNA modifications and non-coding RNA have been the most extensively characterized. The epigenetic machinery modulates DNA accessibility and the recruitment of DNA-binding factors, thus playing pivotal roles in nearly all chromatin-templated cellular processes, such as transcription, DNA replication, DNA damage repair, and chromatin organization. On the one hand, epigenetic factors wrap around the genomic DNA, serving as a mediator of gene-environment interactions, as well as a protective layer against external and internal stimuli, which ensures high-fidelity genetic duplication and DNA repair. On the other hand, chromatin represents a structural barrier that limits the exposure and accessibility of the genomic DNA to various protein complexes, which is precisely regulated to permit or restrict certain DNA-based transactions. Therefore, the dynamic and reversible nature of epigenetic modifications allows for a certain degree of phenotypic plasticity, while preserving genomic integrity.

Histones, a group of positively charged proteins, facilitate the packaging of the eukaryotic genome into a more condensed and manageable structure, known as nucleosomes, the basic repeating units of the chromatin. Each nucleosome consists of a histone octamer, with two copies of the core histones H2A, H2B, H3, and H4, around which approximately 147 base pairs of DNA are wound in a left-handed superhelical turn. Histones undergo extensive post-translational modifications (PTMs), some of which play crucial roles in altering DNA-histone interactions and local chromatin environment, including methylation, acetylation, phosphorylation, etc (Bannister and Kouzarides, 2011; Millán-Zambrano et al., 2022). As an integral component of the epigenetic code, different histone marks are believed to convey specific messages, while the collective PTMs form an intertwined molecular network, charting the chromatin into distinct functional domains (Zhou et al., 2011). With more than 100 different types reported, histone modifications are dynamically regulated by a plethora of catalyzing (writers) and removing (erasers) enzymes, and interpreted by a dedicated group of proteins that can recognize these chemical tags through specific domains (readers). Remarkably, these enzymes can modify both histones and non-histone proteins, thus expanding the regulatory reach of the epigenetic apparatus. Vazquez et al. unveiled the interplay between Sirtuin 7 (SIRT7), an NAD+-dependent histone deacetylase, and p53, a hallmark tumor suppressor, and the functional relevance in embryonic development and tumor progression. Interestingly, SIRT7 has been shown to regulate p53 activity through p300/CBP-associated factor (PCAF)-mediated degradation of murine double minute (MDM2), a p53 E3 ubiquitin ligase (Lu et al., 2020). González et al. reported a novel role of H2A.Z, a non-canonical variant of histone H2A, in the regulation of pericentric heterochromatin through a complex interplay with heterochromatin protein 1 (HP1). Of note, loss of H2A.Z leads to hyper heterochromatinization and increased genome instability related to centromeric defects. Taken together, these reports support the diverse and sometimes controversial roles of histone modifications and associated factors in chromatin regulation.

DNA methylation is another pillar of the epigenetic paradigm that can be stably inherited across multiple cell divisions. 5-methylcytosine (5mC) and its derivative forms, such as 5-hydroxymethylcytosine (5hmC), are amongst the best characterized epigenetic marks in the eukaryotic system. Once thought to be irreversible, 5mC is dynamically catalyzed by DNA methyltransferases (DNMTs) and actively removed by a group of enzymes including the ten-eleven translocation (TET) family proteins (Wu and Zhang, 2017). By opposing the installment of chromatin activators, or directly recruiting repressive complexes through methyl-binding protein domains, DNA methylation is generally associated with transcriptional silencing. Importantly, the inhibition of heterochromatic repeats by DNA methylation prevents their recombination and translocation, therefore safeguarding genome integrity. Notably, the DNA methylome in cancer cells is characterized by genome-wide hypomethylation associated with genomic instability and promoter hypermethylation associated with silencing of tumor suppressor genes (Esteller, 2007). Over the past decades, our knowledge of DNA methylation has been rapidly advancing in both physiological and pathological settings, thanks in part to technological developments. For example, DNA methylation is extensively integrated into the epigenetic network through crosstalk with histone modifications (Li et al., 2021). Targeting DNA methylation by DNMT inhibitors has shown promising potential in boosting antitumor immunity by activating transposable elements, providing novel therapeutic avenues for cancer treatment (Jones et al., 2016; Jones et al., 2019). Besides cancer, faulty DNA methylation has also been frequently documented in other human diseases, including inflammatory and neurological disorders. Ren et al. provided a comprehensive summary of the roles of DNA methylation in idiopathic pulmonary fibrosis (IPF), revealing the mechanistic understanding of its pathogenesis and theranostic opportunities by targeting DNA methylation.

RNA modification is an emerging aspect of epigenetic regulation, forming the so-called “epitranscriptome” that is critical for proper RNA functions, such as transcription and translation. More than 170 different types of RNA modifications have been identified, which have broad effects on gene expression by regulating the folding, stability and transport of RNA, and its interaction with other proteins (Roundtree et al., 2017; Cappannini et al., 2023). Much like histone and DNA marks, RNA modifications are covalent chemical changes to RNA molecules mediated by a growing fleet of writers and erasers, with a group of reader proteins exerting the downstream effects. For example, N6-methyladenosine (m6A), one of the most prevalent and studied mRNA modifications, is installed by the METTL3/METTL14 methyltransferase complex, which regulates gene expression by affecting various features of RNA metabolism (Fu et al., 2014; Roundtree et al., 2017). Modification of transfer RNA (tRNA), the most heavily modified RNA species, is crucial for accurate and efficient protein synthesis. Aberrant tRNA modifications are closely related to human diseases like mitochondrial dysfunction and cancer, known as “RNA modopathies” (Suzuki, 2021; Delaunay et al., 2024). Zhang et al. summarized the latest progresses in RNA modifications and their functional significance in prostate cancer, with a focus on their effects on key androgen receptor signaling pathways and tumor microenvironment.

In summary, the epigenetic blueprint governs a wide range of normal cellular functions, such as embryonic development and stem cell differentiation. Chen et al. presented a review of the mechanisms of induced pluripotent stem cells (iPSCs) and their applications. iPSC, originally achieved by ectopic expression of four transcription factors, Oct3/4, Sox2, Klf4, and c-Myc (OSKM), known as “Yamanaka factors”, can also be obtained by chemical reprogramming targeting epigenetic factors (Hou et al., 2013; Xu et al., 2015; Takahashi and Yamanaka, 2016). Deregulation of the epigenetic apparatus is increasingly recognized as a key player and even one of the drivers of numerous human diseases, presenting both challenges and novel therapeutic strategies for clinical intervention. In recent years, the understanding of chromatin-based epigenetic inheritance mechanisms, such as nucleosome assembly and parental histone segregation, has further demonstrated the intimate coupling of epigenetic maintenance and genetic integrity (Du et al., 2022). Consequently, mutations of parental histone chaperones lead to loss of heterochromatin silencing, defective DNA damage repair, aberrant lineage specification, cell fate alterations and tumor progression. These reports, together with the studies discussed above, underscore the need for a more systematic examination of this essential and expansive molecular network.
